# Supplementary figures and images for: Comprehensive Profiling of Mutations to Influenza Virus PB2 That Confer Resistance to the Cap-Binding Inhibitor Pimodivir
Source: Viruses. 2021 Jun 22;13(7):1196. doi: 10.3390/v13071196 (PMC8310130; doi:10.3390/v13071196)

Figure S2

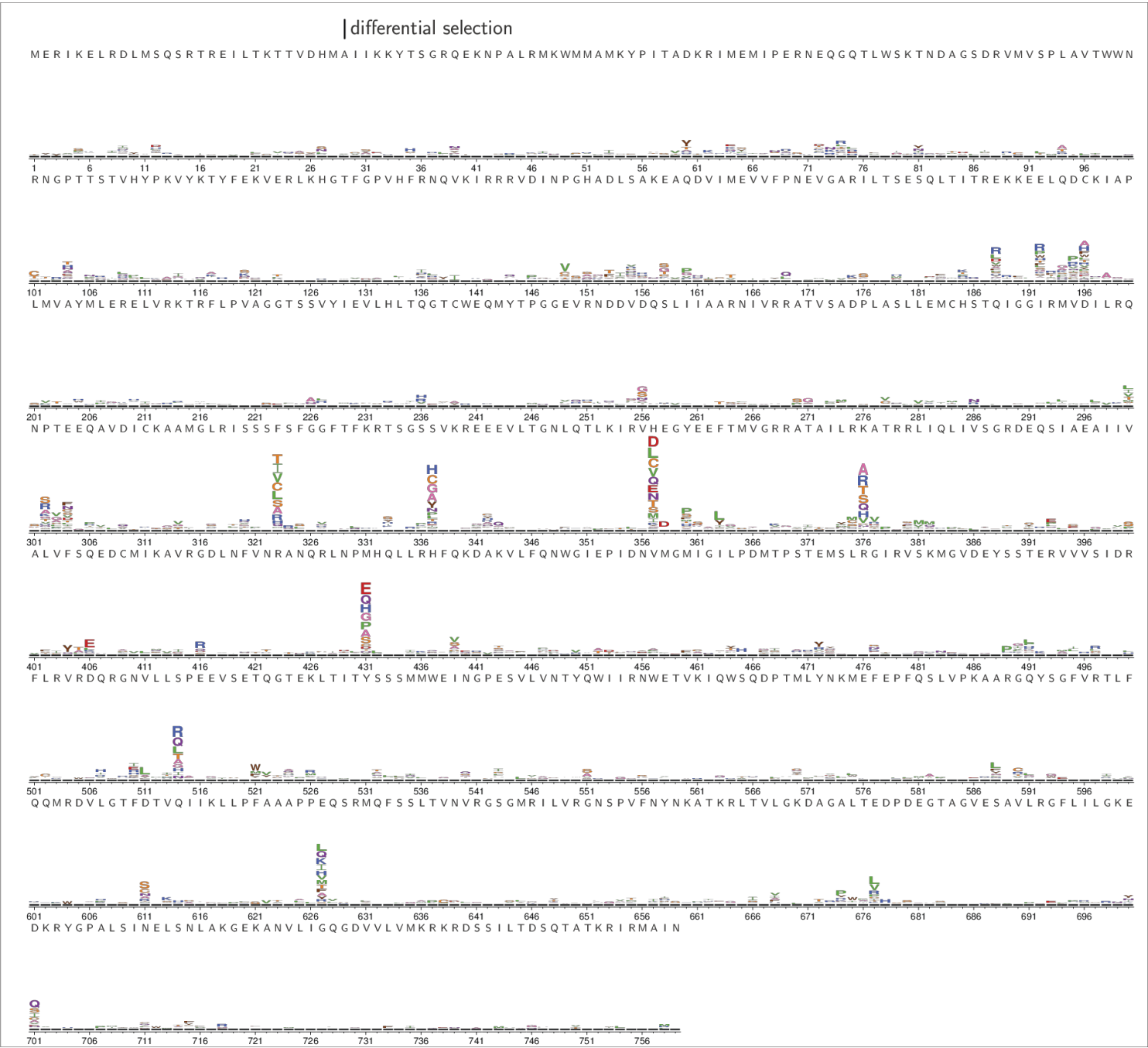

Supplement: Supplementary file 1 [file viruses-13-01196-s001.zip › Supplementary/FigS2.pdf]

Figure S3

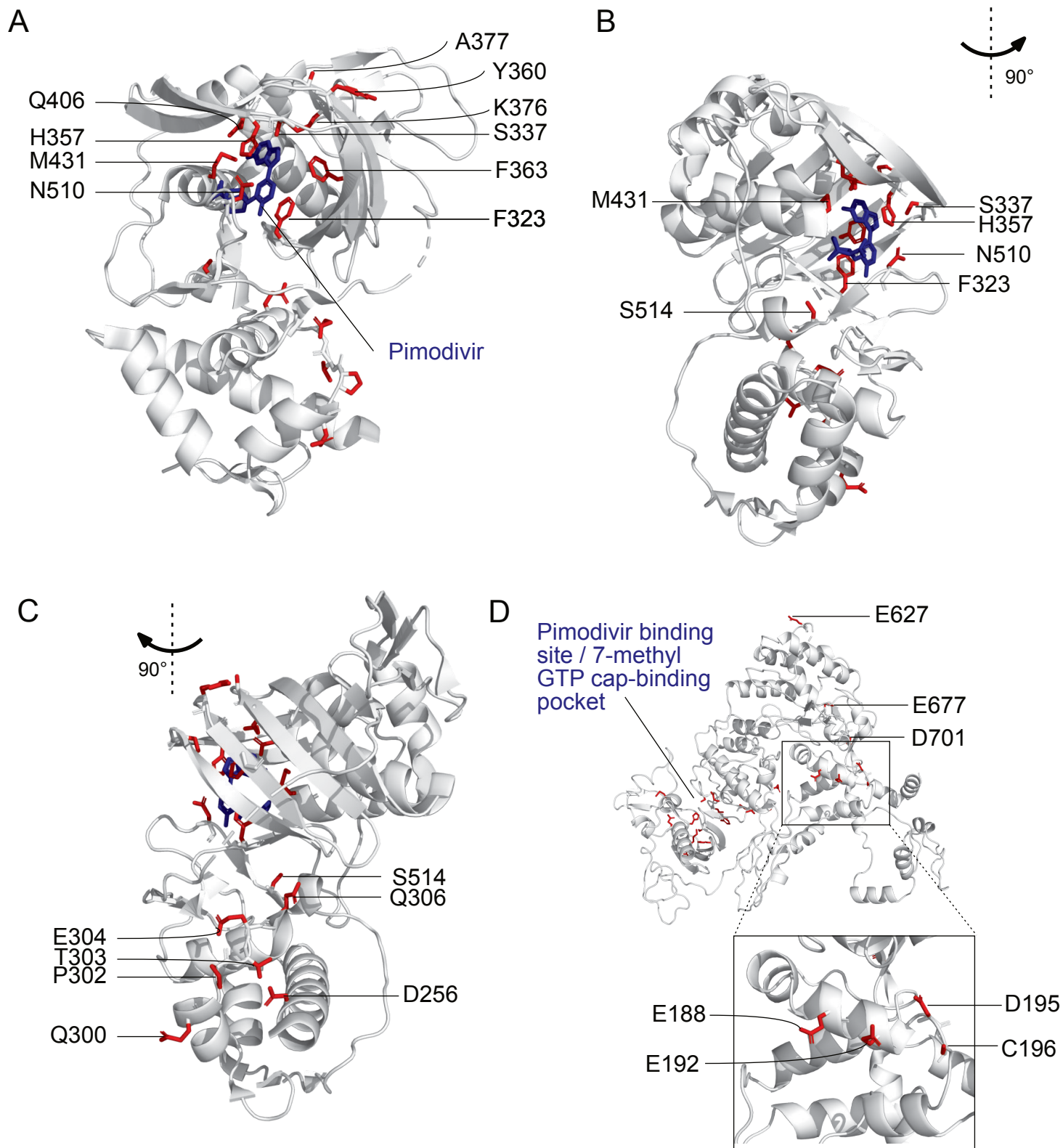

Supplement: Supplementary file 1 [file viruses-13-01196-s001.zip › Supplementary/FigS3.pdf]

Figure S1

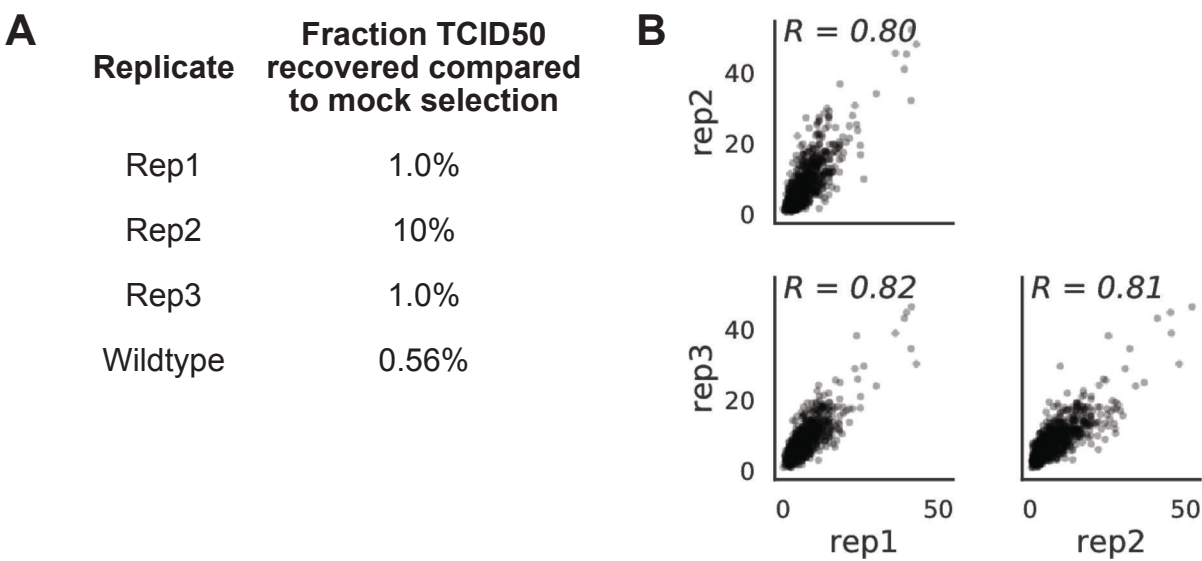

Supplement: Supplementary file 1 [file viruses-13-01196-s001.zip › Supplementary/FigS1.pdf]
